# BNT162b2 COVID-19 Vaccination and Its Effect on Blood Pressure

**DOI:** 10.3390/medicina58121789

**Published:** 2022-12-05

**Authors:** Toh Leong Tan, Sharifah Azura Salleh, Zuraidah Che Man, Michelle Hwee Peng Tan, Rashid Kader, Razman Jarmin

**Affiliations:** 1 Department of Emergency Medicine, Faculty of Medicine, Universiti Kebangsaan Malaysia, Bandar Tun Razak, Kuala Lumpur 56000, Malaysia; 2 Department of Medical Microbiology and Immunology, Faculty of Medicine, Universiti Kebangsaan Malayia, Bandar Tun Razak, Kuala Lumpur 56000, Malaysia; 3 Pharmacy Department, Hospital Canselor Tuanku Muhriz, Universiti Kebangsaan Malaysia, Bandar Tun Razak, Kuala Lumpur 56000, Malaysia; 4 Staff Polyclinic, Hospital Canselor Tuanku Muhriz, Universiti Kebangsaan Malaysia, Bandar Tun Razak, Kuala Lumpur 56000, Malaysia; 5 Department of Surgery, Faculty of Medicine, Universiti Kebangsaan Malaysia, Bandar Tun Razak, Kuala Lumpur 56000, Malaysia

**Keywords:** BNT162b2 vaccine, blood pressure changes, blood pressure monitoring, COVID-19

## Abstract

*Background and Objectives*: The objective of this study is to examine the effect of the BNT162b2 vaccine on systolic blood pressure (SBP), diastolic blood pressure (DBP), mean arterial pressure (MAP), and pulse pressure (PP) before and 15 min after two doses that were given 21 days apart. *Materials and Methods*: This active surveillance study of vaccine safety was conducted on 15 and 16 March (for the first dose) and 5 and 6 April (for the second dose) 2021 in an academic hospital. For both doses, SBP, DBP, MAP, and PP levels were measured before and 15 min after both doses were given to healthcare workers over the age of 18. The results of the study were based on measurements of the mean blood pressure (BP), the mean changes in BP, and the BP trends. *Results*: In total, 287 individuals received the vaccine. After the first dose, 25% (*n* = 72) of individuals had a decrease in DBP of at least 10 mmHg (mean DBP decrease: 15 mmHg, 95% CI: 14–17 mmHg), and after the second dose it was 12.5% (mean DBP decrease: 13 mmHg, 95% CI: 12–15 mmHg). After the first dose, 28.6% (*n* = 82) had a PP that was wider than 40 mmHg. After the first dose, 5.2% and 4.9% of the individuals experienced an increase or decrease in SBP, respectively, of more than 20 mmHg. After the second dose, the SBP of 11% (*n* = 32) decreased by at least 20 mmHg. *Conclusions*: Improved understanding of vaccine effects on BP may help address vaccine hesitancy in healthcare workers.

## 1. Introduction

With the release of the genetic sequence of the SARS-CoV-2 virus, various types of candidate vaccines were developed [1] and approved under the clause of Emergency Use Authorization on 11 December 2020 [2], including the BNT162b2 COVID-19 vaccine. Following the implementation of vaccination, reports of adverse events for immunization (AEFIs) after the first dose of the BNT162b2 vaccine emerged [3]. Any medical event that occurs after immunization can be classified as an adverse event following immunization (AEFI), whether or not the event is related to receiving the immunization [4]. The vaccine responses (product-related, quality defect-related, or error-related), immunization anxiety-related responses, and coincidental events were all included in the AEFIs [4]. Not all AEFIs have a causal relationship with the usage of the vaccine. Because AEFIs may affect a healthy individual after vaccination, prompt action is required to manage it and prevent the public from losing confidence in the vaccination program [5].

There was a recently published case series on eight vaccinees who experienced elevated blood pressure (BP) after receiving the vaccine [6]. Another three studies showed that the BNT162b2 COVID-19 vaccine influenced the blood pressure of vaccinees [7,8,9]. This observation is a valid concern and warrants further investigation of the relationship between the vaccine and BP levels. This information can be useful for the next phase of the COVID-19 vaccination program, especially since it involves subjects with comorbidities.

The BNT162b2 COVID-19 vaccine comprises mRNA that encodes the spike proteins of SARS-CoV-2. Spike proteins are synthesized in the cytoplasm and then expressed on the cell surface [10]. These spike proteins are recognized as foreign bodies by the immune system and are then destroyed. The freely circulating spike proteins in the blood [10,11] will interact with angiotensin-converting enzyme 2 receptor, resulting in a rapid decrease in angiotensin levels [10,11]. This process causes an imbalance between angiotensin II inactivation and the decrease in angiotensin, which may cause blood pressure changes in vaccinees [12,13].

Thus, in this study, we aim to report on the database created from our vaccination safety active surveillance (VSAS) on the BP trend before and 15 min after vaccination. We aim to explore the trends of systolic BP (SBP), diastolic BP (DBP), mean arterial pressure (MAP), and pulse pressure (PP) before and 15 min post the first and second doses of the BNT162b2 vaccine. We hypothesize that the BNT162b2 vaccine is associated with BP changes before vaccination and 15 min after vaccination.

## 2. Materials and Methods

Active surveillance was carried out as part of COVID-19 vaccination program activities to monitor BNT162b2 vaccination safety among our hospital healthcare workers (HCW). All vaccine-related data that were collected were kept in a secured location and only accessible to authorized personnel. The VSAS was set up by the Vaccination Committee of Hospital Canselor Tuanku Muhriz (HCTM) under the patronage of the Hospital Director, following reports of BP fluctuation and other adverse events post-vaccination in other vaccination centers across the world. This BNT162b2 COVID-19 VSAS program was carried out on 15–16 March 2021 for the participants’ first dose and on 5–6 April 2021 for the second dose, which meant that the doses were administered 21 days apart. The surveillance was carried out at the designated vaccination center in HCTM, the teaching hospital of Universiti Kebangsaan Malaysia (UKM), located in Cheras, Kuala Lumpur, Malaysia. 

### 2.1. Study Subjects’ Eligibility

Subjects included in the surveillance were healthcare staff currently working in HCTM, aged above 18 years, and they received their doses as scheduled. All subjects with any comorbidities were also included in the study. Subjects with missing data on blood pressure were excluded. Any subject who had dropped out for the second dose of the vaccine was excluded from the analysis.

### 2.2. Data Collection

The protocol for this surveillance was integrated into the existing vaccination protocol, which consists of pre-vaccination health screening for premorbid conditions and history of allergic reactions. The vaccine recipients were asked if they had common comorbidities such as hypertension, diabetes, cardiovascular disease, stroke or transient ischemic attack, asthma, and others. Allergy history was also surveyed. The BP of vaccinees was measured pre- and 15 minutes post-vaccination. A standardized form was created to record age, gender, comorbidities, and pre- and post-vaccination BP measurements including any adverse reactions. During the observation period, any recipient noted to have high BP or AEFI/s was referred to the on-site staff clinic and the Emergency Department if deemed necessary.

### 2.3. Measuring of Blood Pressure 

The pre-vaccination BP measurement was recorded by trained personnel for each patient in the sitting position after a 5 min rest using a digital BP-measuring machine (Connex ProBP 3400) with appropriately sized cuffs. Post-vaccination BP measurement was recorded 15 min after compulsory resting and observation. Two measurements were taken for all vaccinees. In those who recorded a BP difference of more than 10 mm Hg for either systolic or diastolic BP, the third measurement would be repeated after a 5 min rest. The final recorded BP measurements were the mean of the second and third recorded measurements. Vaccinees with SBP ≥140 mmHg had their heart rates assessed. If they were found to be tachycardic with a heart rate of ≥100 beats/min, they were asked to rest for 5 min, and thereafter have their heart rate re-measured. Vaccinees were allowed to receive their injections if they were no longer tachycardic after resting. Otherwise, their appointment would be rescheduled. For this group of vaccinees, the final recorded BP was the measurement taken after rest.

### 2.4. Definition of AEFIs

All healthcare workers experiencing side effects were interviewed over the phone 72 h after vaccination. Pain at the injection site is defined as pain at the injection site within 72 h of injection. Numbness over the injection site is defined as pins and needles over the injection site within 72 h post-injection. Fatigue is defined as feeling tired or sleepy within 72 h post-injection. Giddiness is defined as any light-headedness occurring within 72 h post-injection. Headache was defined as any episode of pain over the head that occurred within 72 h post-injection. Nausea is defined as any episode of nausea that occurs within 72 h post-injection. Near-fainting is defined as any episode of near-blackout within 72 h post-injection. Difficulty breathing is defined as self-reported difficulty breathing within 72 h post-injection. Body itchiness is defined as itchiness occurring over any part of the body within 72 h post-injection.

### 2.5. The BNT162B2 COVID-19 Vaccine

The BNT162b2 COVID-19 vaccines comprise lipid nanoparticles that contain mRNA. These lipid nanoparticles are immediately taken up by host cells after being injected into the muscle. The mRNA enters the cytosol of the host cell and directs the production of the SARS-CoV-2 spike protein. The spike protein is then expressed on the host cell’s surface. The expansion of neutralizing antibodies against the SARS-CoV-2 protein-specific T cell (CD4+ and CD8+) is then induced [14].

### 2.6. The HCTM Vaccination Protocol

Vaccine recipients were notified of their appointments via the *Mysejahtera* application, which is an online phone application created by the Malaysian government as one of the means to monitor COVID-19 infection and track COVID-19 vaccination progress. On the date of the vaccination appointment, the vaccinees confirmed their attendance by scanning the QR code of the vaccination site using *Mysejahtera*. A medical officer was on site to answer any queries from the recipient. Once written consent was obtained for vaccination, recipients were allocated to a designated area while awaiting their vaccination. Recipients would proceed to the vaccination booth when their turn arrived. In the vaccination booth, a staff nurse would check the identity of the recipient and explain each step of the vaccination process to the recipient. The vaccine was then administered, and the recipients were asked to rest in a resting area for a minimum of 15 min while being observed for any acute side effects. Once the observation was completed, the recipients were given some general advice about monitoring future side effects. A vaccine card was issued to the vaccinees upon completion of the first dose and electronic certificates were issued upon completion of the second dose. 

### 2.7. Statistical Analysis Plan

#### 2.7.1. Sample Size

This is a report on our hospital’s VSAS, where the sampling method was to actively include all consecutive subjects systematically from surveillance records. The sample size was calculated based on the interim data from our COVID-19 VSAS database. The sample size was calculated based on an effect size of 0.2, with a 2-sided *p*-value less than 0.05. The total sample size required to achieve a statistically significant difference with a mean power of 80% was 199 paired subjects. 

#### 2.7.2. Data Analysis

Data analysis was carried out using IBM SPSS for Windows (version 25.0). For descriptive analysis, participants’ characteristics and other categorical variables were expressed in frequency and percentages. Those who received both doses of vaccines were subjected to analysis. Analysis of participant age depended on the normal distribution and was presented as the mean or median with corresponding standard deviation (SD) or interquartile range, and 95% confidence interval. Further analysis of the comparison between means was performed using Student’s *t*-test. Paired *t*-test was used for within-group comparison. All tests were considered significant at *p* < 0.05. Multiple imputation analysis was used to analyze missing data when the significance was more than 5%. A difference of SBP ≥ 20 mmHg (SBP pre-vaccination—SBP post-vaccination) and DBP ≥ 10 mmHg was considered significant. PP ≥ 40 mmHg was classified as wide pulse pressure. Any difference of PP ≥ 10 mmHg (PP pre-vaccination—PP post-vaccination) was considered a significant change. For BP trends, ‘elevated’ and ‘decreased’ trends were defined as an increased or decreased mean difference of ≥20 mmHg for SBP, ≥10 mmHg for DBP, and ≥10 mmHg for PP between pre-vaccination and 15 min after vaccination. Normal range BP was recorded as ‘no change’. High blood pressure is defined as a BP increase of 20 mmHg from baseline.

### 2.8. Ethical Approval

The study was approved by UKM Human Ethical Review Board (JEP-2021-302). The active surveillance was carried out as part of COVID-19 vaccination program activities to monitor BNT162b2 vaccination safety among our hospital healthcare workers (HCW). Due to the retrospective nature of this study, it waived the need for informed consent.

## 3. Results

### 3.1. Study Population

After screening our VSAS database, a total of 288 vaccinees who received their first dose of vaccine on 15–16 March 2021 fulfilled the criteria. Twenty-one days later, on 5–6 April 2021, this same cohort completed their second dose. At the same time, the study also achieved its targeted sample size of 199 subjects. During the data analysis, we excluded one subject due to missing BP values, which contributed to 0.3% of the missing data. There were 12 subjects (2.8%) with missing BP data in the first-dose analysis. The missing data were considered completely missing at random, which required no further imputation analysis. The age of the study group ranged from 21 to 60 years. The demographic and clinical characteristics of the study population for the analysis are shown in Table 1. The study inclusion flow is shown in Figure S1. A total of eight participants (2.8%) with hypertension were included in this surveillance. Details of their reports are shown in Supplementary file Table S1 (Supplementary Materials). There were no severe adverse events from the COVID-19 vaccination or death 15 min after receiving dose 1 and dose 2 vaccination during this surveillance period. Details of AEFIs are shown in Supplementary file Table S2 (Supplementary Materials). Among the AEFIs reported by vaccinees were pain over the injection site (dose 1 = 18.1% and dose 2 = 20.9%), numbness over the injection limbs (dose 1= 11.8% and dose 2 = 7.3%), fatigue (dose 1= 6.3% and dose 2 = 1.4%), giddiness (dose 1 = 3.8% and dose 2 = 7.7%), nausea (dose 1= 1.4% and dose 2 = 1.0%), near-fainting (dose 1= 0.3% and dose 2 = 0.3%), difficulty of breathing (dose 1 only = 0.3%), and body itchiness (dose 2 only = 0.3%). No AEFIs were associated with an increase in SBP or DBP either after dose 1 or dose 2 vaccination (*p* > 0.005, Table S2). 

### 3.2. Trends and Means of SBP, DBP, and PP Changes between Pre- and 15-Minute Post-BNT162B2 Vaccination in the Analysis

We analyzed the trend of BP changes for those who completed both doses of vaccines. The results are shown in Table 2. The percentages of vaccinees with elevated or decreased mean SBP of 20 mmHg or more were 5.2% and 4.9%, respectively, 15 min after first-dose vaccination. For the elevated SBP group (*n* = 14), the mean SBP change was 25 mmHg (95% CI: 23–28 mmHg), while the decreased SBP group (*n* = 15) had a mean SBP change of 27 mmHg (95% CI: 23–30 mmHg). Nevertheless, upon receipt of the second dose in the same cohort, an increased percentage of vaccinees (11%) had a decrease in SBP of ≥20 mmHg with mean SBP changes of 27 mmHg (95% CI: 25–30 mmHg) 15 min post vaccination, compared to 3.1% who had elevated SBP by ≥20 mmHg (mean SBP change: 26 mmHg, 95% CI: 24–28 mmHg). Nevertheless, we concluded that this was not a true SBP reduction. We noted that the pre-vaccination SBP was higher, to begin with, upon the second dose as compared to the first dose.

Interestingly, 15 min after the first dose, one-quarter of vaccinees (25.1%) had a decrease in DBP by ≥10 mmHg compared to only 6.6% who were noted to have elevated DBP by ≥10 mmHg (Table 2). In the group with decreased DBP, the mean BP change was 15 mmHg (95% CI: 14–17 mmHg), while the change in the elevated DBP group was 15 mmHg (95% CI: 13–17 mmHg). Upon receiving the second dose, the percentage of vaccinees with decreased DBP by ≥10 mmHg was also high (12.5%), compared to those who had elevated DBP by ≥10 mmHg (9.1%). DBP change for the decreased group was 13 mmHg (95% CI: 12–15 mmHg) and 15 mmHg for the elevated group (95% CI: 12–19 mmHg). Interestingly, a higher proportion of the vaccinees had reduced DBP by ≥10 mmHg after receiving the first dose compared to the second dose (25.1% vs. 12.5%).

Percentages of vaccinees with widened or narrowed PP were 28.6% (mean PP changes: 17 mmHg, 95% CI: 16–19 mmHg) and 12.2% (mean PP changes: 15 mmHg, 95% CI: 13–16 mmHg), respectively, comparing between before and 15 min after the first dose of vaccination (Table 2). However, upon receiving the second dose, there was a paradoxical observation in terms of PP trends compared to the first dose. After the second dose, the percentage of vaccinees who had narrowed PP (21.6%, mean PP changes: 18 mmHg, 95% CI: 16–21 mmHg) almost doubled, while the percentage of vaccinees with widened PP reduced by almost half (12.9%, mean PP changes: 15 mmHg, 95% CI: 14–17 mmHg). 

### 3.3. Vaccination and Blood Pressure

The data are shown in Table 3. After the vaccinees received their first dose, the mean DBP was observed to decrease by 3 mmHg, the mean MAP was decreased by 2 mmHg, and the mean PP was widened by 3 mmHg at 15 min post-vaccination as compared to pre-vaccination. However, the mean SBP was unchanged for the first dose. The BP analysis for second-dose vaccination showed a reduction in mean SBP of 3 mmHg, a decrease in the mean MAP of 2 mmHg, and a paradoxical trend of the mean PP, which was narrowed by 3 mmHg at 15 min after receiving it. The mean DBP remained the same for the second dose.

## 4. Discussion

Many people are concerned about the possible side effects of COVID-19 vaccination, especially with the newly developed mRNA vaccines, including BNT162b2 from Pfizer which has received EUA. One of the side effects that has caused concern is the effect of the vaccine on BP. In our recent VSAS on the BNT162b2 vaccine, we observed a few minor adverse events, including significant transient fluctuation in BP after vaccination as compared with before vaccination. Although fluctuations in BP are not considered one of the AEFIs, these can significantly affect subjects with comorbidities. This concern seems valid, as there was a recently published case series on eight vaccinees who experienced elevated blood pressure after receiving the vaccine [6].

Based on our VSAS, a few noteworthy findings related to vaccinees’ BP after BNT162b2 vaccination were established. Throughout the surveillance, we noted that there were a few minor AEFIs reported that were generally well tolerated by the vaccinees. Among all vaccinees who received the first dose of this vaccine, one-quarter of vaccinees experienced a transient decrease in DBP and one-third of them displayed widened PP. However, this phenomenon was not observed upon the second dose of vaccination. Interestingly, mean SBP remained unchanged after the first dose of the vaccine, with minimal reduction after receiving the second dose. These findings of mean DBP reduction and mean PP widening after the first vaccination have not been reported elsewhere. 

The findings of lower DBP and widened PP after the first dose of the vaccine were unexpected. There are limited reports on transient effects compared to the long-term effect of DBP fluctuation. A study by Jaakko et al. found that patients with pre-existing stage II and III hypertension whose DBP was below 90 mmHg had a higher risk of dying from cardiovascular disease compared with patients with mean DBP in the range of 90–109 mmHg [15]. The same study also concluded that lower DBP among patients with WHO stage I hypertension was not significantly associated with cardiovascular disease mortality. The study also found that significant low DBP among pre-existing cardiac failure patients may increase the risk of cardiovascular death. The study also highlighted the increased risk of mortality for hypertensive patients above 50 years old with lower DBP. This study concluded that patients who had comorbid conditions such as neurovascular disease, ischemic heart disease, and cancer with mean DBP below 90 mmHg were found to be associated with higher mortality than those with DBP in the range of 90–109 mmHg. A study by Witteman et al. suggested that the decrease in DBP indicates vessel wall stiffening and parallels the degree of atherosclerosis progression [16].

This transient BP fluctuation after vaccination may present a hypothetical risk of vaccinees developing neurovascular [17,18,19] and cardiovascular adverse events [20], especially for those who have comorbid diseases [21,22,23]. No neuro- or cardiovascular events occurred in our active surveillance cohort, implying that the risk of these adverse events occurring due to the vaccination may be minimal. Our cohorts were rather young, and the majority were free of comorbidities. Furthermore, our cohorts were mainly HCWs and had easy access to the hospital facility. In the event of any major fluctuation in BP, they would have been immediately monitored at the on-site staff clinic or referred to the Emergency Department. Those who have comorbidities should therefore be closely monitored after the vaccination. A pre-vaccination blood-pressure check and a 15-minute post-vaccination check may help to reduce the risk of these complications, especially in those with comorbid conditions.

On the other hand, our surveillance found that 10% of vaccinees were observed to have lower SBP after receiving the second dose of vaccine as compared to the first dose. However, this was not observed during the first dose of vaccination, which prompts the question of whether it was directly related to the vaccine or the psychology of vaccinees. Furthermore, we were unable to rule out whether this transient hypertension was related to white coat syndrome. We suspected that the vaccinees may have been anxious about the hype surrounding the higher AEFI risk after the second dose, thus leading to higher pre-vaccination SBP [24]. This percentage of increased pre-SBP may seem to be small, but its effect on stroke or cardiovascular events for healthy vaccinees is unclear [25]. Bouhanick et al. pointed out that 31% of those with grade III hypertension after the first injection were still hypertensive at the same stage after the second injection [7]. About 15% of the patients who were normotensive after the first injection had high BP after the second one. The findings are different from our results. Our study found that 5% of subjects had elevated SBP, but only 3% persisted with higher SBP after the second dose of the vaccine. The mean age of the study population for this paper was older (59 years old ± 20) as compared to our population’s median age, which is younger (33 years old, IQR 31–39). It is a known fact that the vascular walls are more compliant in the younger population than in the older population, which may lead to more effective blood pressure regulation and hemostasis in the younger group. Our finding is similar to what Sanidas et al. found [8]. Their study suggests that there were short-term changes in blood pressure after receiving the mRNA-based vaccination, which supports our study results, even though the mean age for the study population was higher than our study.

Zappa et al. created an online survey that reported that 6 out of 113 subjects (5.3%) showed an average rise in systolic or diastolic BP 5 days before and after the first dose of Pfizer/BioNTech vaccine [9]. Two of the subjects (1.78%) with a BP rise after the first dose also experienced a BP rise after the second dose. We reported 5–7% of elevated SBP and DBP after the first dose of the vaccine, and the findings are similar. However, 3% and 9% persisted with high SBP and DBP, respectively, after the second dose. Our study found a higher percentage of decreased SBP and DBP after the second dose of vaccination, which was not noted by Zappa et al.

We also found that about one-third of the vaccinees had wide pulse pressure after receiving the first dose of the vaccine. The widened pulse pressure and lower DBP after the first dose of the vaccine may indicate the presence of vasodilation. This vasodilation may be caused by a mild anaphylactic reaction possibly related to vaccines or their components. PP is the pulsatile component of repetitive continuous waves produced by BP that propagates along the arterial tree [26]. An increase in PP of 10 mmHg was found to increase the risk of a cardiovascular event, stroke, or overall mortality by 10–20%. A few studies suggested that PP may be a better predictor of cardiovascular events among the middle-aged population [27,28,29]. However, there was no report associating transient changes in PP with vaccine-related adverse events. 

The VSAS program was formed to monitor the safety of HCWs in HCTM. The surveillance actively monitors our center’s vaccinees’ status and looks out for AEFI. The committee actively reports, manages, and monitors the vaccinees in the event of AEFI cases. Having a VSAS program ensures that potential AEFIs are promptly treated; this directly improves the confidence of vaccinees toward the COVID-19 vaccination program. 

Overall, this surveillance provides a clearer picture of vaccine-related blood-pressure changes, particularly for healthcare workers. Vaccine apprehension is widespread, particularly among younger female healthcare workers [30]. Improved understanding of vaccine side effects may alleviate vaccine hesitancy among healthcare workers [31].

The strength of this study lies in its active surveillance design. The timing of BP measurements (pre- and 15-minutes post-vaccination) was within the acceptable time frame to directly examine vaccine-related adverse events. This study has a few limitations. First, it was a single-center study, and the participants were all healthcare workers, who may not be representative of the general population. The length of time used to track the subjects’ progress may be insufficient to determine the full extent of the harm caused by temporary BP fluctuations. The use of antihypertensive drugs, thyroxine, or caffeine by the vaccinees was another potential confounder for the BP increases that was not accounted for in the surveillance.

## 5. Conclusions

Our surveillance found that the BNT162b2 vaccination may be associated with transient changes in blood pressure. Our VSAS findings may provide information that fills a gap in understanding the effects of BNT162b2. 

## Figures and Tables

**Table 1:** Demographic and Clinical Characteristics of Participants Who Received Both Doses of BNT162b2 Vaccine.

Demographic Characteristics	Number of Vaccinees (N = 287)
**Age, years, median (IQR)**	33 (31–39)
**Age group, years, n (%)**	
21–29	58 (20.2)
30–39	165 (57.5)
40–49	54 (18.8)
50–60	10 (3.5)
**Gender, n (%)**	
Male	76 (26.5)
Female	211 (73.5)
**Comorbidities, n (%)**	45 (15.7)
Asthma	9 (3.1)
HTN	8 (2.8)
Diabetes Mellitus	7 (2.4)
Ischemic Heart Disease	3 (1.0)
Allergy	2 (0.7)
Renal Disease	1 (0.3)
Others	15 (5.2)

**Table 2:** BP Trends, Mean Changes between Pre- and Post-BNT162b2 Vaccination.

	1st Dose (*n* = 287)					2nd Dose (*n* = 287)				
Blood Pressure Trends	Frequency, *n* (%)	BP Changes, Mean, mmHg	Standard Deviation, mmHg	Standard Error	95% Confidence Interval, mmHg	Frequency, *n* (%)	BP Changes Mean, mmHg	Standard Deviation, mmHg	Standard Error	95% Confidence Interval, mmHg
**Systolic**										
Elevated *	15 (5.2)	25	5	1.33	23 to 28	9 (3.1)	26	3	1.08	24 to 28
Decreased ^†^	14 (4.9)	27	6	1.69	23 to 30	32 (11.1)	27	7	1.20	25 to 30
No Change ^‡^	258 (89.9)	0	9	1	−1 to 1	246 (85.7)	1	9	0.58	−1 to 2
**Diastolic**										
Elevated *	19 (6.6)	15	5	1.10	13 to 17	26 (9.1)	15	9	1.64	12 to 19
Decreased ^†^	72 (25.1)	15	6	0.68	14 to 17	36 (12.5)	13	6	0.97	12 to 15
No Change ^‡^	196 (68.3)	1	5	0.34	1 to 2	225 (78.4)	0	5	0.2	−1 to 1
**Pulse Pressure**										
Widened	82 (28.6)	17	8	0.81	16 to 19	37 (12.9)	15	6	0.93	14 to 17
Narrowed	35 (12.2)	15	5	0.77	13 to 16	62 (21.6)	18	9	1.17	16 to 21
No Change	170 (59.2)	−1	5	0.38	−1 to 1	188 (65.5)	1	12	0.36	1 to 4

* SBP, DBP, and PP increased by ≥20 mmHg, ≥10 mmHg, and ≥10 mmHg, respectively. † SBP, DBP, and PP decreased by ≥20 mmHg, ≥10 mmHg, and ≥10 mmHg, respectively. ‡ SBP, DBP, and PP were within the normal range.

**Table 3:** Comparison of BP Measurements Pre- and 15-minutes Post-BNT162b2 Vaccination in the Analysis.

Vaccine Doses		1st Dose (*n* = 287)			2nd Dose (*n* = 287)		
		Mean, mmHg (SD)	95% Confidence Interval, mmHg	*p*-Value (Student’s *t*-Test)	Mean, mmHg (SD)	95% Confidence Interval, mmHg	*p*-Value (Student’s *t*-Test)
Systolic	pre-vaccination	125 (15)	123 to 127	1.00	127 (18)	125 to 129	0.001 *
	post-vaccination	125 (16)	123 to 127		124 (17)	122 to 126	
Diastolic	pre-vaccination	81 (10)	80 to 83	<0.001 *	80 (10)	79 to 82	0.55
	post-vaccination	78 (11)	76 to 79		80 (11)	79 to 81	
Mean arterial pressure	pre-vaccination	96 (11)	95 to 97	0.003 *	96 (12)	95 to 97	0.025 *
	post-vaccination	94 (12)	91 to 94		95 (12)	94 to 96	
Pulse pressure	pre-vaccination	44 (11)	42 to 45	<0.001 *	47 (12)	45 to 48	<0.001 *
	post-vaccination	47 (12)	46 to 49		44 (11)	42 to 46	

* *p*-value of <0.05 is considered statistically significant.

## Data Availability

All vaccine-related data that were collected were kept in a secured location and only accessible to authorized personnel. The data that support the findings of this study are available from Toh Leong Tan. However, restrictions apply to the availability of these data, which were used under license for the current study, and so are not publicly available. Data are, however, available from the authors upon reasonable request and with the permission of Toh Leong Tan.

## Supplementary Materials

The following are available online at https://www.mdpi.com/article/10.3390/medicina58121789/s1, Figure S1: Study population for BNT162b2 vaccines; Table S1: Detail Demography of the Vaccinees Who Had a History of Hypertension under Treatment in Both-Group Analysis; Table S2. Frequencies of Adverse Events for Immunization After Both Doses of Vaccinations and Their Association with Blood Pressure.
