# Supplementary material for: BNT162b2 COVID-19 Vaccination and Its Effect on Blood Pressure
Source: Medicina (Kaunas). 2022 Dec 5;58(12):1789. doi: 10.3390/medicina58121789 (PMC9786719; doi:10.3390/medicina58121789)
Supplement: Supplementary file 1 [file medicina-58-01789-s001.zip › medicina-1957659-SI.pdf]

Data Supplement

BNT162b2 COVID-19 VACCINATION AND ITS EFFECT ON BLOOD PRESSURE

Toh Leong Tan[1*,#a], Sharifah Azura Salleh[2,#a], Zuraidah Che Man[1,#a], Michelle Hwee Peng Tan[3,a], Rashid Kader[4,#a], Razman Jarmin[5,#a].

1. Department of Emergency Medicine, Faculty of Medicine, Universiti Kebangsaan Malaysia
2. Department of Medical Microbiology and Immunology, Faculty of Medicine, Universiti Kebangsaan Malaysia
3. Pharmacy Department, Hospital Canselor Tuanku Muhriz, Universiti Kebangsaan Malaysia
4. Staff Polyclinic, Hospital Canselor Tuanku Muhriz, Universiti Kebangsaan Malaysia
5. Department of Surgery, Faculty of Medicine, Universiti Kebangsaan Malaysia

#a Hospital Canselor Tuanku Muhriz, Universiti Kebangsaan Malaysia, 56000 Cheras, Kuala Lumpur, Malaysia.

*Corresponding Author

Email: sebastianttl@yahoo.co.uk

CONTENT

List	Content	Page
**Figure S1**	Study population for BNT162b2 vaccines.	3
**Table S1**	Detail Demography of the Vaccinees Who Had a History of Hypertension	4
**Table S2**	Frequencies Of Adverse Events for Immunisation After Both Doses of Vaccination and Their Association with Blood Pressure	5

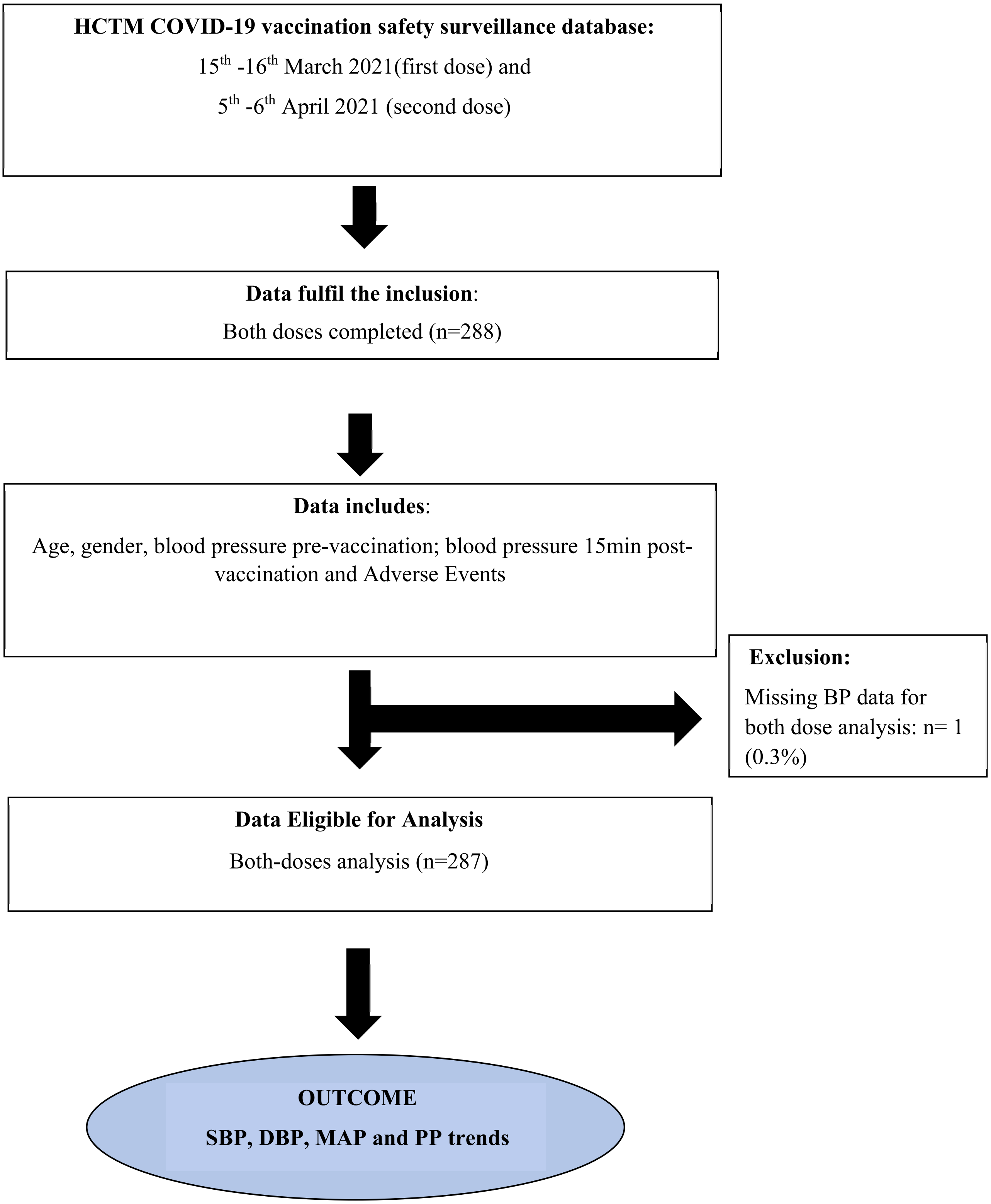

Figure S1. Study population for BNT162b2 vaccines.

Table S1. Detail Demography of the Vaccinees Who Had a History of Hypertension under Treatment

Age, years	Gender	First Dose of Vaccine Blood Pressure, mmHg				Second Dose of Vaccine Blood Pressure, mmHg			
		Pre-vaccination SBP	Pre-vaccination DBP	Post-vaccination SBP	Post-vaccination DBP	Pre-vaccination SBP	Pre-vaccination DBP	Post-vaccination SBP	Post-vaccination DBP
56-60	Male	187	78	152	54	164	78	164	77
46-50	Male	151	94	151	96	172	99	174	98
46-50	Female	140	71	150	93	139	93	133	96
41-45	Female	159	91	156	95	114	73	124	79
56-60	Female	155	87	156	85	145	83	145	89
41-45	Female	145	91	149	84	136	81	124	77
41-45	Female	108	79	103	71	140	79	144	105
41-45	Female	134	88	130	86	139	90	134	91

Table S2. Frequencies Of Adverse Events for Immunisation After Both Doses of Vaccination and Their Association with Blood Pressure

	Adverse Event for Immunisation (n=287)	Dose 1, n (%)	Systolic increase more than 20mmHg, P-value	Diastolic increase more than 10mmHg, P-value	Dose 2, n (%)	Systolic increase more than 20mmHg, P-value	Diastolic increase more than 10mmHg, P-value
1	Pain over the Injection Site	52 (18.1)	0.47[a]	0.46[a]	60 (20.9)	0.28[a]	0.83[b]
2	Numbness over the Injection Site	34 (11.8)	0.27[a]	0.40[a]	21 (7.3)	0.50[a]	0.13[a]
3	Fatigue	18 (6.3)	0.63[a]	0.66[a]	4 (1.4)	0.88[a]	0.68[a]
4	Giddiness	11 (3.8)	0.11[a]	0.54[a]	22 (7.7)	0.52[a]	0.62[a]
5	Headache	5 (1.7)	0.76[a]	0.71[a]	4 (1.4)	0.88[a]	0.68[a]
6	Nausea	4 (1.4)	0.80[a]	0.24[a]	3 (1.0)	0.91[a]	0.75[a]
7	Near Fainting	1 (0.3)	0.95[a]	0.07[a]	1 (0.3)	0.97[a]	0.91[a]
8	Difficulty in Breathing	1 (0.3)	0.95[a]	0.07[a]	0 (0)	-	-
9	Body Itchiness	0 (0.0)	-	-	1(0.3)	0.97[a]	0.09[a]
10	No AEFI	161(56.1)	0.45[b]	0.07[b]	171 (59.6)	0.27[a]	0.20[b]

[a] Fisher's Exact Test

[b] Chi's Square Test

*p-value less than 0.05 is considered statistically significant.
